# Tobacco (*Nicotiana tabacum*) PSY peptides and their potential roles in seed germination, vegetative growth, and leaf senescence under osmotic stress

**DOI:** 10.3389/fpls.2025.1575308

**Published:** 2025-05-28

**Authors:** Zhichao Deng, Xiaolu Pan, Rongrong Wu, Yalun Yang, Tao Liu, Wei Li, Zenglin Zhang, Yongfeng Guo

**Affiliations:** ^1^ Tobacco Research Institute, Chinese Academy of Agricultural Sciences, Qingdao, China; ^2^ Institute of Industrial Crops, Anhui Academy of Agricultural Sciences, Hefei, China; ^3^ College of Agronomy, Qingdao Agricultural University, Qingdao, China

**Keywords:** PSY peptides, tobacco, secreted peptides, gene expression patterns, plant development, osmotic stress

## Abstract

**Introduction:**

Members of the Plant Peptides Containing Sulfated Tyrosine (PSY) family play critical roles in plant development and stress responses. While extensively studied in Arabidopsis, rice, and wheat, the biological functions of PSY peptides in tobacco (*Nicotiana tabacum*) remain poorly characterized. This study aims to identify *NtPSY* genes in tobacco and elucidate their roles in growth regulation and osmotic stress adaptation.

**Methods:**

A comprehensive bioinformatics approach was employed to identify *NtPSY* genes using tobacco genomic data and homology with *Arabidopsis* PSY sequences. Expression profiles under drought, salinity, and temperature stress were analyzed via qRT-PCR. Functional validation included virus-induced gene silencing (VIGS) of homologous genes in *Nicotiana benthamiana* and exogenous application of synthetic NtPSY1/NtPSY3 peptides to assess their effects on seed germination, seedling growth, and osmotic stress tolerance.

**Results:**

A total of nine *NtPSY* genes were identified, the expression changes of which under different abiotic stresses were analyzed using qRT-PCR. The results showed that all *NtPSY* genes responded significantly to drought, salinity, and extreme temperature stress conditions. NtPSY1 and NtPSY3 were further analyzed for their function in development and response to osmotic stress. The results indicated that treatments of tobacco detached leaves and seeds with synthetic peptides can promote seed germination and seedling growth, while reducing tobacco’s tolerance to osmotic stress.

**Discussion:**

The dual role of NtPSY peptides—promoting growth under optimal conditions while impairing stress tolerance—highlights their function as signaling molecules balancing growth-stress trade-offs. This mechanism aligns with PSY receptor-mediated pathways reported in Arabidopsis, where ligand binding inhibits stress signaling. The study provides novel insights into PSY peptide dynamics in tobacco and suggests potential applications for optimizing stress resilience in crops.

## 1 Introduction

More and more secreted peptides have been characterized as crucial signaling molecules that facilitate cell-to-cell communication in plants, orchestrating and delineating cellular functions (Matsubayashi, 2014). Secreted peptides play vital roles in numerous aspects of plant developmental processes and responses to biotic and abiotic stressors (Tavormina et al., 2015). They perform various functions, including regulating cell division and differentiation, guiding organ development, and mediating stress responses. Most ligands function by binding to and activating specific receptors and downstream signaling pathways (Butenko et al., 2009; Matsubayashi, 2011; Czyzewicz et al., 2013), though some, such as PSY peptides, modulate cellular responses by inhibiting receptor activities. Moreover, peptides as signaling molecules exhibit high specificity and flexibility, allowing them to rapidly respond to environmental changes and regulate the physiological status of plants (Stuhrwohldt and Schaller, 2019). Structurally, secreted peptide signals can be categorized into two primary groups: post-translationally modified peptides (PTM) and cysteine-rich peptides (CRP) (Matsubayashi, 2011).

PTM peptides are secreted only after undergoing modification and processing within the secretory pathway, and with common modifications including tyrosine sulfation, proline hydroxylation, and hydroxyproline arabinosylation (Tavormina et al., 2015; Matsubayashi, 2018). PSY peptides are a type of PTM peptides requiring sulfation, and their subcellular localization is extracellular (Matsubayashi, 2011). Sulfation modification plays a crucial role in the biological activity of PSY peptides, ensuring that these peptides can accurately recognize their specific receptors in the extracellular environment and effectively mediate signal transduction (Moore, 2003). AtPSY1, a member of the PSY family in *Arabidopsis*, was the first characterized PSY member. It is an 18-amino acid peptide in its mature form that undergoes post-translational modifications including tyrosine sulfation and hydroxyproline arabinosylation (Amano et al., 2007). Besides PSY, plants produce three other types of tyrosine-sulfated peptides: phytosulfokines (PSKs) (Matsubayashi and Sakagami, 1996), root meristem growth factors (RGFs) (Matsuzaki et al., 2010), and Casparian strip integrity factors (CIFs) (Doblas et al., 2017; Nakayama et al., 2017). PSKs, RGFs, and CIFs have been characterized to be involved in various aspects of plant development, growth, and stress responses (Kaufmann and Sauter, 2019). Therefore, these peptides exert their biological functions through sulfation modification, which is a key mechanism in plant normal growth, development, and response to environmental changes.

Recent studies have revealed the significant role of PSY peptides in plant growth, development, and stress responses. Currently, nine, eight, and twenty-nine *PSY* genes have been identified in *Arabidopsis thaliana*, rice (*Oryza sativa* L.), and wheat (*Triticum aestivum* L.) (Pruitt et al., 2017; Tost et al., 2021; Kesawat et al., 2024). Among them, detailed functional studies have been particularly conducted on AtPSY1. It has been shown to promote root growth (Amano et al., 2007; Pruitt et al., 2017), increase cell size, facilitate seedling cuticle development (De Giorgi et al., 2021), and activate plasma membrane H^+^-ATPase AHA2 through phosphorylation (Fuglsang et al., 2014). The receptors for AtPSY1 and other AtPSYs have been identified as PSY receptors (PSYRs). In the absence of PSY peptide ligands, the PSYRs inherently induce stress-responsive genes and restrict growth. In the presence of the ligands, the receptors are inactivated such that the stress-responsive signaling is inhibited, thereby promoting growth (Ogawa-Ohnishi et al., 2022). This ligand-deprivation-dependent activation system may enable plants to modulate the balance between growth and stress responses. Furthermore, PSY peptides enhance disease resistance by regulating the plant immune system. The PSY1-like sulfated peptide RaxX was also identified in *Xanthomonas oryzae*, the pathogen of rice. RaxX triggers rice’s immune response by directly binding to the immune receptor XA21 (Pruitt et al., 2015; Luu et al., 2019). This mechanism further highlights the importance of PSY peptides in plant physiological regulation.

Tobacco ranks among the world’s foremost cash crops (Budzianowski, 2015). However, recent climatic shifts have intensified drought conditions, significantly impeding tobacco cultivation (Hussain et al., 2019; Chaudhry and Sidhu, 2022). Developing drought-resistant varieties can not only bolster the survival and yield of tobacco in arid regions but also stabilize the incomes of farmers involved in its cultivation (Setiawan et al., 2022). Furthermore, as a model plant, research on the mechanisms of drought tolerance in tobacco has provided valuable genetic insights for other crops, potentially enhancing the resilience and adaptability of agriculture (Gubiš et al., 2007; Rabara et al., 2015). In this study, we identified nine *PSY* genes in tobacco based on homology with the sequences of nine AtPSY peptides from *Arabidopsis.* We performed comprehensive bioinformatics analysis and experimental validation to discover the expression of *NtPSY* under different abiotic stresses, and investigated the functions of *NtPSY1* and *NtPSY3* by transient silencing experiments. The results showed that exogenously applied synthesized mature PSY peptides increased the sensitivity of isolated leaves to osmotic stress and improved tobacco seed germination and seedling growth, but under osmotic stress conditions, PSY peptides treatments instead impaired seed germination and seedling growth. These findings reveal the potential role of PSY peptides in tobacco growth, development, and stress response, and provide new insights for further understanding of the biological processes involved in plant growth and stress response. This study focuses on NtPSY (Plant Peptides Containing Sulfated Tyrosine), which, although sharing the same abbreviation with Protein NtPSY (Phytoene Synthase), is a distinct entity with different properties.

## 2 Materials and methods

### 2.1 Plant materials, growth conditions and stress treatments

Common tobacco (*Nicotiana tabacum*) variety K326 was used in this study. Tobacco seeds were first sterilized and then sown on Murashige and Skoog (MS) solid medium. After germination, seedlings were grown in a controlled environment until they developed four true leaves. These seedlings were then transferred to MS liquid medium to serve as the control group. To simulate drought stress, seedlings were transferred to MS liquid medium supplemented with 300 mmol/L mannitol (Zhu, 2002). For salt stress treatments, the MS liquid medium was amended with 100 mmol/L NaCl. Cold stress was simulated by maintaining plants at 4°C, and heat stress was imposed by exposing them to 42°C. Sampling occurred at 0, 1, 3, and 6 hours post-treatment, with six plants from each treatment group harvested and immediately preserved in liquid nitrogen for subsequent analyses.

### 2.2 Identification and sequence characterization of *PSY* gene family members in tobacco

To identify all members of the PSY gene family in common tobacco, protein and nucleic acid sequences were retrieved from the Solanaceae Genome Database (https://solgenomics.net/). Concurrently, PSY protein sequences from *Arabidopsis* were sourced from the TAIR database (https://www.arabidopsis.org). Following multiple sequence alignment using CLUSTAL 2.1, a Hidden Markov Model (HMM) was constructed for the conserved domain of *Arabidopsis* PSY proteins. Subsequently, the hmmsearch program was employed to query the tobacco protein database, employing an E-value threshold of 1e^-20^. Identified candidates were used in multiple sequence alignment to construct a tobacco-specific HMM for the conserved domain of PSY proteins. Using HMMER, a subsequent search was conducted on tobacco nucleic acid sequences, employing an E-value threshold of 0.01. Redundant sequences were filtered from the resultant dataset, with the remaining sequences classified as members of the tobacco PSY gene family.

Using the online tool ProtParam from Expasy (https://web.expasy.org/protparam/), the physical and chemical properties such as length, molecular weight, isoelectric point, aliphatic index (AI), and grand average of hydropathy (GRAVY) of tobacco PSY family proteins were calculated. Signal peptide prediction was conducted using the online tool SignalP 6.0 (https://services.healthtech.dtu.dk/services/SignalP-6.0/). The NtPSY was named based on their positions in the phylogenetic tree.

### 2.3 Phylogenetic and structural analyses of PSY gene family

The PSY protein sequences for *Arabidopsis*, rice (*Oryza sativa*), maize (*Zea mays*), soybean (*Glycine max*), and chickpea (*Cicer arietinum*) will be downloaded from the UniProt database (https://www.uniprot.org). Phylogenetic trees will be constructed using MEGA11 (https://www.megasoftware.net/) for the selected species and tobacco PSY proteins. Sequence alignment will be performed with the ClustalW method, explicitly excluding non-conserved regions outside the aligned domains. The phylogenetic tree will be constructed using the Maximum Likelihood method, with a bootstrap value of 1000.

### 2.4 Gene structure and conserved motif analysis

The online tool MEME (https://meme-suite.org/meme/) (Bailey et al., 2009) was used to predict conserved motifs in the NtPSY proteins, with motif length set to 6-100 and a maximum of 10 motifs to be identified, while keeping other parameters at their default values. Analysis of gene structure was based on the genomic DNA and CDS sequences of tobacco PSY gene family members, and the gene structures were visualized using the online Gene Structure Display Server 2.0 (https://gsds.gao-lab.org/Gsds_help.php).

### 2.5 Chromosomal location and cis-acting regulatory element analysis

Chromosomal positions of *PSY* genes and the lengths of tobacco chromosomes from the tobacco genome annotation file (GFF file) were extracted, and the chromosomal locations of *PSY* family members were visualized using TBtools (https://github.com/CJ-Chen/TBtools) (Chen et al., 2020). The 2000 bp upstream region from the start codon of each *PSY* gene in the tobacco genome sequence file was determined, followed by an analysis of the cis-acting regulatory elements (CAREs) in these promoter regions using the PlantCARE software (http://bioinformatics.psb.ugent.be/webtools/plantcare/html/) (Lescot et al., 2002). Cis-acting elements that are commonly found in all plants were excluded, and the remaining cis-acting elements were categorized into three major classes based on their functions. Finally, all identified elements in the promoter regions of *NtPSY* genes were presented in the form of a heatmap by tbtools (Chen et al., 2020).

### 2.6 Expression pattern analysis of *NtPSYs* in different tissues

From the tobacco gene expression atlas published by EDWARDS in 2010 (http://www.ebi.ac.uk/arrayexpress/experiments/E-MTAB-176/) (Edwards et al., 2010), data from 19 different tissues including cotyledon, seed, flower, open bud, closed bud, cauline leaf, late senescent leaf, mid/late senescent leaf, mid/early senescent leaf, early senescent leaf, mature leaf, young leaf, upper stem, lower stem, floral shoot apex, vegetative shoot apex, young shoot, mature root, and young root were selected. These samples cover the entire lifecycle from seed germination to plant senescence. The sample identifiers matching the tobacco PSY gene family were retrieved, and the corresponding gene expression profile data was obtained using these sample identifiers.

### 2.7 Protein tertiary structure and subcellular localization prediction

Tertiary structures of tobacco PSY proteins were constructed using the online website I-TASSER (http://zhanggroup.org/I-TASSER) (Zhou et al., 2022), and visual analysis and annotation of cleavage sites marked as XX/DYXXXG (cleavage sites shown by ‘/’) were performed using the software PyMOL.The subcellular localization prediction was performed using the ProtComp v.9.0 software available on the online website Softberry (http://linux1.softberry.com).

### 2.8 RNA extraction and real-time PCR analysis

Total RNA was extracted from stress-treated samples using the Ultrapure RNA kit (CW biotech, Beijing, China). Subsequently, 1 µg of RNA was reverse transcribed into cDNA using the HiScript III All-in-one RT SuperMix (Vazyme, NanJing, China). The cDNA was diluted 20-fold and used as the template for Real-time PCR. The PCR program was set as follows: initial denaturation at 95°C for 10 s; followed by 40 cycles of 95°C for 10 s and 60°C for 30 s. The melting curve analysis was conducted with the following steps: 95°C for 15 s, 60°C for 60 s, and 95°C for 15 s.

Data were calculated and analyzed using the 2^-ΔΔCT^ method (Rao et al., 2013), with *NtActin* as the reference gene. The results were compared and integrated with other data. Significance analysis was performed using the ANOVA method with a significance level set at 0.05. The primers used in this study are listed in **Table 1**, and each sample was tested in triplicate.

**Table 1:** Detailed characteristics of tobacco PSYs.

Protein ID	Assigned Name	Location Start point	Location End point	Chromosome	AA	CDS	Molecular Weight	Theoretical pI	Signal Peptide	GRAVY	Aliphatic Index	Subcellular Localization
Nitab4.5_0001128g0100.1	*NtPSY1*	4451459	4451941	Chr01	91	276	10.38	6.22	YES	-0.222	83.63	Extracellular
Nitab4.5_0006000g0100.1	*NtPSY2*	154288	155075	Nitab4.5_0006000	89	270	10.17	6.34	YES	-0.303	77.750	Extracellular
Nitab4.5_0008269g0020.1	*NtPSY3*	59397983	59898336	Chr03	81	246	8.98	7.73	YES	-0.090	88.020	Extracellular
Nitab4.5_0000121g0170.1	*NtPSY4*	51659535	51660541	Chr24	86	261	9.65	5.46	YES	-0.117	86.280	Extracellular
Nitab4.5_0002028g0030.1	*NtPSY5*	40362044	40362598	Chr20	70	213	7.69	6.8	YES	-0.510	73.860	Extracellular
Nitab4.5_0000011g0030.1	*NtPSY6*	82081027	82081594	Chr24	72	219	8.06	6.23	NO	-0.329	81.250	Extracellular
Nitab4.5_0007090g0010.1	*NtPSY7*	55835614	55836163	Chr01	83	252	9.40	6.51	YES	-0.278	88.070	Extracellular
Nitab4.5_0000548g0010.1	*NtPSY8*	43109006	43109943	Chr04	77	234	8.76	10.59	YES	-0.264	92.340	Extracellular
Nitab4.5_0005491g0040.1	*NtPSY9*	8202096	8202644	Chr19	88	267	10.05	11.03	YES	-0.108	92.950	Extracellular

### 2.9 Silencing of *PSY* genes in *Nicotiana benthamiana* using VIGS

The tobacco PSY proteins were divided into five groups based on sequence similarity. TRV2 vectors for virus-induced gene silencing (VIGS) were constructed using homologous regions of *Nicotiana benthamiana PSY* genes, with *TRV2::PDS* as a positive control. Two-week-old uniformly grown *N. benthamiana* plants were co-infiltrated with a 1:1 mixture of *TRV2::PSYs* and *TRV1* (10 plants/group). When PDS-injected plants showed photobleaching after 10 days, healthy leaves from equivalent positions of the five *TRV2::PSY* groups were collected (6 plants/group). Leaf discs (16/group) of uniform size were treated with 300 mmol/L mannitol for osmotic stress (three independent replicates). Chlorophyll content and ion leakage were measured with three biological replicates (each containing three technical replicates). Statistical differences were determined by Duncan’s test (p< 0.05), with letters indicating significance.

### 2.10 Measurements of chlorophyll content and ion leakage

For chlorophyll content determination (Guo and Gan, 2006), the weight of each group of leaf disks was recorded as M, and the disks were immersed in 10 mL of absolute ethanol (V). The samples were decolorized under dark conditions at room temperature. Once completely decolorized, 200 μL of each sample was transferred to a 96-well plate. The absorbance at 665 nm and 649 nm was measured using a microplate reader (with absolute ethanol as a reference), denoted as A665 and A649, respectively. The chlorophyll content was calculated using the following formulas:


Ca=13.95 ∗ A665–6.88 ∗ A649

Cb=24.96 ∗ A649–7.32 ∗ A665

Total chlorophyll content (mg/g)=(Ca+Cb) ∗ V/W

For ion leakage measurement, each group of leaf disks was immersed in deionized distilled water and shaken in a 25°C water bath for 30 minutes. Electrical conductivity was measured using a digital conductivity meter. The samples were then boiled for 10 minutes, and conductivity was monitored again. The percentage difference between the initial and final conductivity readings was used as an indicator of membrane leakage (He and Gan, 2002).

### 2.11 Functional characterization of PSY peptides under osmotic stress conditions

The peptide sequences of NtPSY1 and NtPSY3 were submitted to GenScript Biologicals (Nanjing, China) for *in vitro* synthesis, with sulfation modification at the N-terminal tyrosine residue and a purity requirement >95%. The synthesized NtPSY peptides were designed as follows: NtPSY1: D{TYR(SO3)}PGSGANNRHMPRPQL GR; NtPSY3: D{TYR(SO3)}PGAGANNHHTPGHP.

The NtPSYs peptides were dissolved and diluted in double-distilled water to prepare 1 mM stock solutions based on their molecular weights. For experimental treatments, the peptide stock solutions were added to both standard MS solid medium and MS medium supplemented with 300 mM mannitol, achieving a final working concentration of 1 μM NtPSYs peptides. After germination on MS solid medium, tobacco seedlings were transferred to MS media with or without PSY peptides, and with or without mannitol supplementation. Each group contained 6 seedlings with three biological replicates. After 7 days of treatment, root length and leaf area were measured using the ImageJ software (https://imagej.nih.gov/ij/), and the number of lateral roots was recorded. Statistical analysis was performed using the t-test to determine whether the differences between groups were statistically significant.

In the germination rate experiment, 100 tobacco seeds per group were placed in water, or in 100 mM mannitol solution, and the above solutions containing NtPSY peptides, with daily observations of seed germination. Leaf discs (16/group) from 6-week-old tobacco plants were punched and placed separately in water containing 1 μM PSY peptides, 300 mM mannitol solution, and control treatments without PSY peptides (three independent replicates). After 8 days, Chlorophyll content and ion leakage were measured with three biological replicates (each containing three technical replicates). Statistical differences were determined by Duncan’s test (p< 0.05), with letters indicating significance.

## 3 Results

### 3.1 Identification and annotation of *NtPSYs*

Through two rounds of HMM build and search, nine members of the PSY gene family in tobacco were identified. Analysis of the conserved protein domains confirmed their classification within the PSY gene family, and based on subsequent evolutionary analysis they are designated as *NtPSY1-9*. Protein physicochemical properties were computed using the ProtParam tool on Expasy. The NtPSY proteins exhibit lengths varying from 70 amino acids (NtPSY5) to 91 amino acids (NtPSY1), molecular weights from 7.69 kDa (NtPSY5) to 10.38 kDa (NtPSY1), and isoelectric points from 5.46 (NtPSY4) to 11.03 (NtPSY9). Their negative GRAVY values indicate a hydrophilic nature. Based on the Aliphatic Index, all members except NtPSY2 (77.750) and NtPSY5 (73.860) have values exceeding 80, suggesting higher stability, particularly under elevated temperatures. In addition, the subcellular localization predictions of all NtPSYs are extracellular (**Table 1**).

### 3.2 Systematic phylogenetic, gene structure, and chromosomal localization analysis of *NtPSYs*

**Figure 1:**
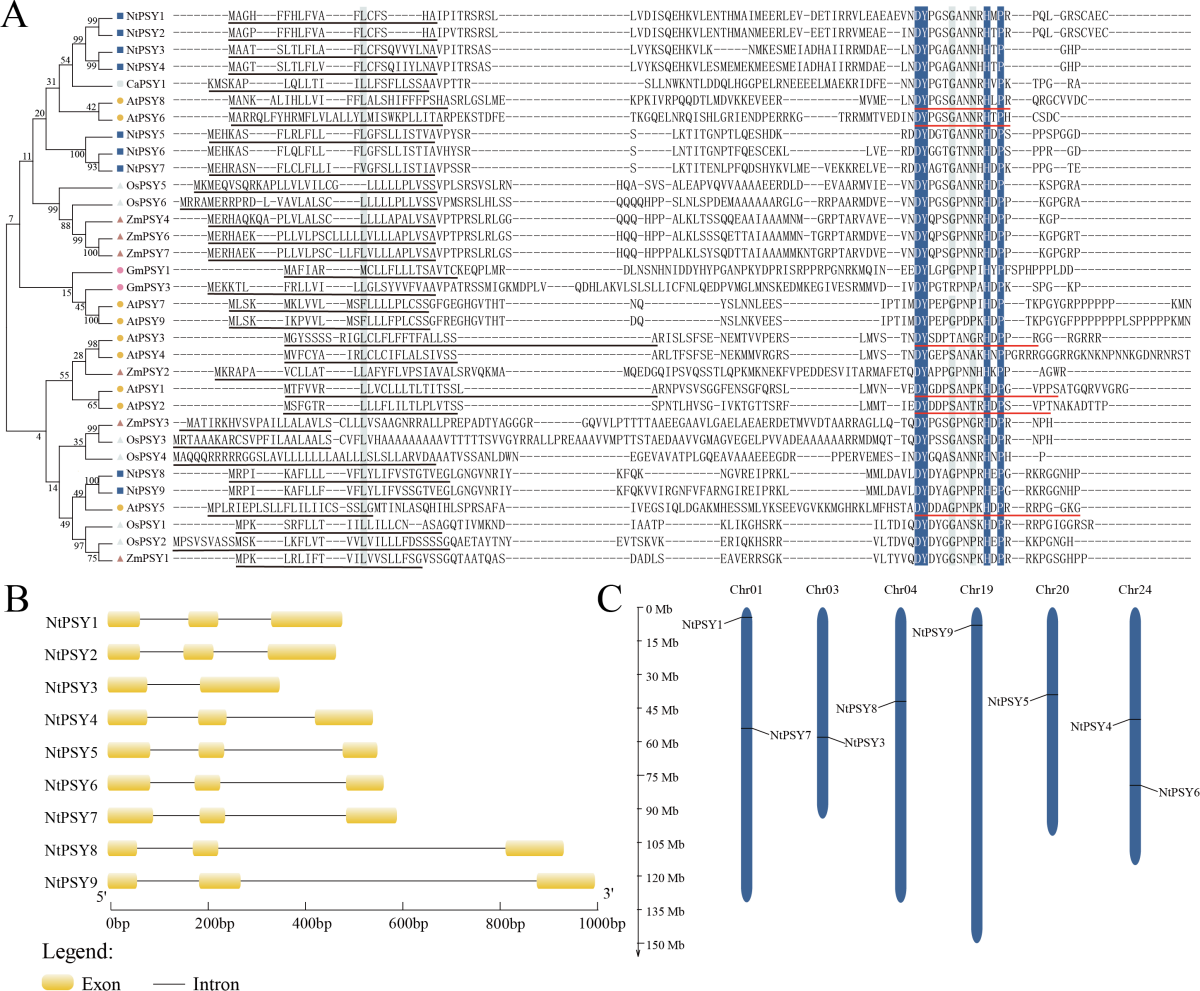
Identification, localization, and structural analysis of the tobacco *PSY* gene family. **(A)** Phylogenetic tree of the tobacco *PSY* family members with PSY proteins from other plant species, constructed using MEGA 11.0 software, employing the Maximum Likelihood (ML) method. Sequence alignment with PSY proteins from other species is represented. Conserved amino acid residues are indicated by blue boxes. Mature PSY peptide sequences identified in Arabidopsis are marked with red underlines, while predicted signal peptides are indicated with black underlines. **(B)** Exon-intron structures of *NtPSY* genes. Coding sequences (CDS) are depicted by yellow boxes, and introns are represented by black lines. Exon and intron lengths are shown according to the scale at the bottom. **(C)** Chromosomal localization of *NtPSY* genes, with chromosome lengths indicated by the scale on the left side.

To delve into the evolutionary relationships of NtPSYs, we constructed a phylogenetic tree using protein sequences from various species (**Figure 1A**). The tree revealed that the NtPSY gene family members segregate into two main subgroups, with NtPSY1-7 belonging to the first subgroup and NtPSY8-9 to the second. The signal peptide is a short peptide chain that directs newly synthesized proteins towards the secretory pathway. Sequence alignment of PSY proteins from different species (**Figure 1A**) indicated that all tobacco PSY proteins except NtPSY6 possess signal peptides. NtPSY6, akin to NtPSY5 in protein structure, likely underwent mutations during evolution, resulting in the loss of its signal peptide due to changes in two amino acids. Furthermore, precursor peptide sequences of PSY exhibit substantial variability across species, whereas PSY structural domains remain conserved. These conserved domains harbor five key amino acids—DY, N, H, and P—where tyrosine serves as a crucial site for sulfate modification. The conservation of these domains across multiple plant species underscores the pivotal role of the PSY family in plant development.

To analyze gene structure of the NtPSY family, we examined the exon-intron distribution within the coding sequences of the 9 *NtPSY* genes (**Figure 1B**). Except for *NtPSY3*, which contains two introns, all other genes in this family have three introns, indicating a conservative gene structure among tobacco *PSY* genes. Utilizing annotation data from the Solanaceae genomic database, we mapped the genomic locations of the 9 *NtPSY* genes (**Figure 1C**). Visualization showed that the *NtPSY* genes are unevenly distributed across six chromosomes: two genes each on Chr1 and Chr24, and one gene each on Chr3, Chr4, Chr19, and Chr20. Notably, *NtPSY2* was not anchored to any contigs, primarily due to incomplete whole-genome sequencing of tobacco.

### 3.3 Analysis of tissue-specific expression and promoter regions of the *NtPSYs*

**Figure 2:**
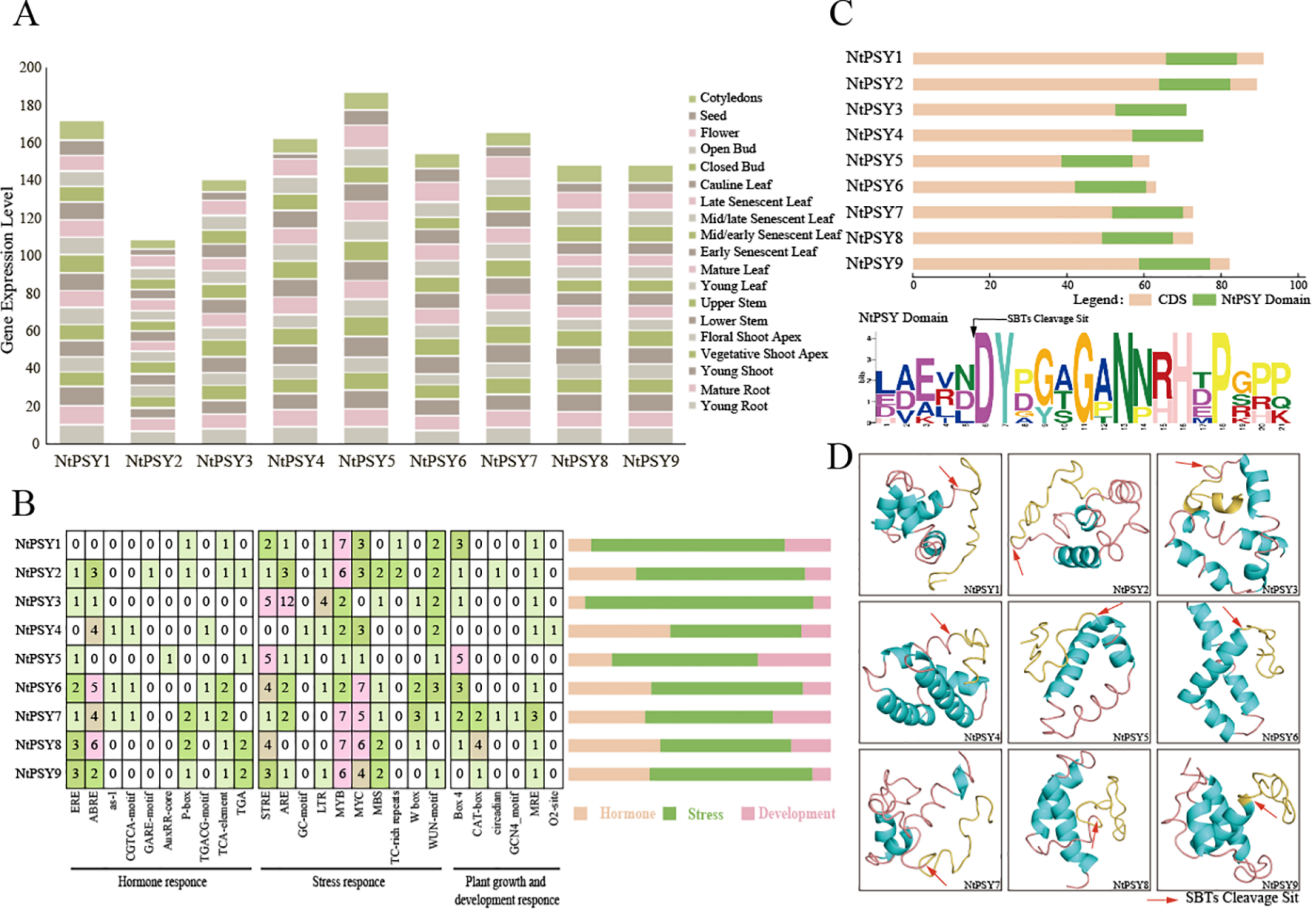
Expression patterns, cis-acting elements, and protein structure of NtPSYs. **(A)** Expression patterns of *NtPSYs* in cotyledon, seed, flower, open bud, closed bud, cauline leaf, late senescent leaf, mid/late senescent leaf, mid/early senescent leaf, early senescent leaf, mature leaf, young leaf, upper stem, lower stem, floral shoot apex, vegetative shoot apex, young shoot, mature root, and young root. **(B)** Common cis-regulatory elements in the upstream 2kb promoter sequence of the *NtPSY* genes. **(C)** Conservation domain analysis of NtPSY proteins. **(D)** Tertiary structure of NtPSY proteins, where blue indicates α-helices, yellow represents the mature PSY sequence, and red arrows indicate the SBT processing sites. Hormone response element abbreviations/functions: ERE, Ethylene responsive element; ABRE, Abscisic acid responsive element; as-1, Activator sequence 1; CGTCA-motif, Cytokinin responsive element; GARE-motif, Gibberellic acid responsive element; AuxRR-core, Auxin responsive element core; P-box, G-box; TGACG-motif, MeJ responsive element; TCA-element, Salicylic acid responsive element; TGA, -motif-like sequence stress response element abbreviations/functions: STRE, Stress responsive element; ARE, Anaerobic responsive element; GC-motif, Gibberellin-responsive element; LTR, Low temperature responsive element; MYB, MYB binding site involved in drought-inducibility; MYC, MYC binding site involved in drought-inducibility; MBS, MYB binding site involved in drought-inducibility; TC-rich repeats, cis-acting element involved in defense and stress responsiveness; W box, WRKY binding box; WUN-motif, Wound responsive motif Development Response element abbreviations/functions: Box 4, TELO-box (telobox); CAT-box, Cysteine regulatory element; circadian, Circadian clock regulatory element; GCN4_motif, GCN4-like motif; MRE, Metal response element; O2-site, Oxygen responsive element.

Plant peptides function as signaling molecules and are often present in trace amounts in plant tissues, making them difficult to detect. Therefore, the expression levels of genes encoding these peptides are frequently used to assess their presence. To predict the functions of NtPSYs during growth and development, we obtained expression levels of these 9 *NtPSYs* in cotyledon, seed, flower, open bud, closed bud, cauline leaf, late senescent leaf, mid/late senescent leaf, mid/early senescent leaf, early senescent leaf, mature leaf, young leaf, upper stem, lower stem, floral shoot apex, vegetative shoot apex, young shoot, mature root, and young root, which represent 19 tissues spanning the entire life cycle of tobacco, from the Solanaceae genomic database. The expression levels were plotted as a bar graph (**Figure 2A**), and it was observed that expression of the *NtPSYs* were detected in all the tested tissues, and the expression levels were relatively consistent. This implies that the tobacco PSY gene family plays a crucial role throughout the entire developmental process.

To better understand the transcriptional regulation of *NtPSYs*, we obtained the 2000 bp upstream promoters of these genes and searched the PlantCARE database to identify cis-acting elements. As shown in **Figure 2B**, the promoter regions of the *NtPSY* genes contain a diverse array of cis-elements (**Figure 2B**). Among the 26 identified cis-elements, regulatory elements related to abiotic stress, plant hormone responses, and growth regulation—three core physiological processes—were identified. Notably, stress-related elements are abundant in the promoters of all the tobacco *PSY* genes, with MYB and MYC elements nearly universal across all *NtPSY* promoters. Particularly, *NtPSY3* and *NtPSY8* exhibit higher expression levels under various stress conditions such as jasmonic acid and cold stress, indicating their potential roles in plant stress tolerance mechanisms. Additionally, traditional plant hormone regulatory elements are also widespread on the *NtPSY* promoters, suggesting potential cross-regulatory interactions between PSY peptides and classical hormones during tobacco development (**Figure 2B**).

### 3.4 Conserved structure and tertiary structure analysis of NtPSY proteins

Upon analyzing the conserved structural domains of NtPSY proteins (**Figure 2C**), it is evident that all NtPSYs possess a conserved PSY protein domain at their C-terminus. Further examination of the functional domains of NtPSYs reveals conserved amino acid residues D, Y, G, N, H, and P, which are crucial for precursor peptide processing and mature peptide function.

Analysis of the tertiary structure of NtPSY proteins (**Figure 2D**) reveals diverse structural features: NtPSY1 consists of multiple short helices and coiled segments; NtPSY2 exhibits a compact structure with a prominent long helix, several short helices, and additional coiled sections; NtPSY3 features two longer helices and several shorter ones; NtPSY4 is characterized by dense helices and short connecting chains; NtPSY5 includes two main helices linked by longer coiled chains; NtPSY6 comprises closely packed helices and short linking chains; NtPSY7 displays a complex structure with large helices and intricate connections; NtPSY8 contains three main helices with tighter connections and shorter coiled chains; NtPSY9, similar to NtPSY8, has more complex and extended linking chains between helices. Across all these structures, α-helices predominate, but their number, length, and arrangement vary, potentially influencing protein function and stability.

### 3.5 Expression patterns of *NtPSYs* in response to stress treatments

**Figure 3:**
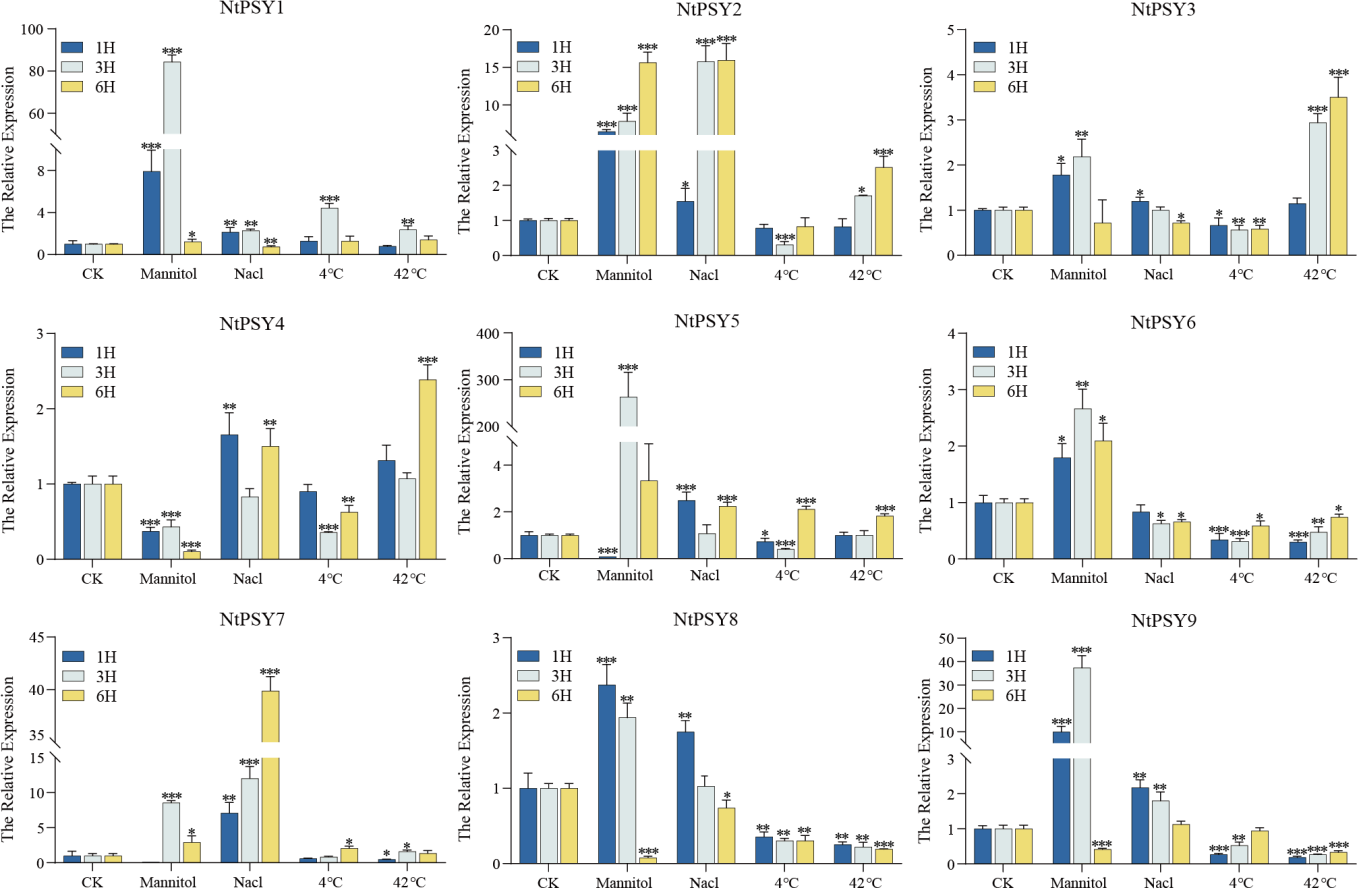
Quantitative real-time PCR (qRT-PCR) analysis of the expression of *NtPSYs* under different stress conditions. The relative abundance of *NtPSYs* transcripts in various treatments was calculated as fold changes relative to control (CK) treatment values, with normalization to *NtActin*. Error bars represent the standard error of the mean (SEM) with n = 3. P< 0.05 (*), P< 0.01 (**), and P< 0.001 (***) (ANOVA).

To analyze the potential function of NtPSYs in stress responses, we examined their expression patterns under four types of stress treatments: osmotic stress, salt stress, low temperature stress, and high temperature stress (**Figure 3**). We found that all *NtPSYs* responded to these stresses, exhibiting varying expression patterns across different stresses and different time points within the same stress condition. Specifically, except for *NtPSY4* which was down-regulated under osmotic stress, all other *NtPSYs* showed up-regulation. Notably, *NtPSY5* was up-regulated by 280-folds after 3 hours of osmotic stress treatment. Under salt stress, all *NtPSYs* except *NtPSY3* and *NtPSY6* were up-regulated. *NtPSY2*, *NtPSY3*, and *NtPSY4* were up-regulated under high temperature stress, whereas most *NtPSYs* showed insensitivity or down-regulation under cold stress. These results suggest that *NtPSY* may play significant roles in the tobacco stress response processes.

### 3.6 Analysis of osmotic stress tolerance in *Nicotiana benthamiana* detached leaves with VIGS-mediated silencing of *NbPSYs*

**Figure 4:**
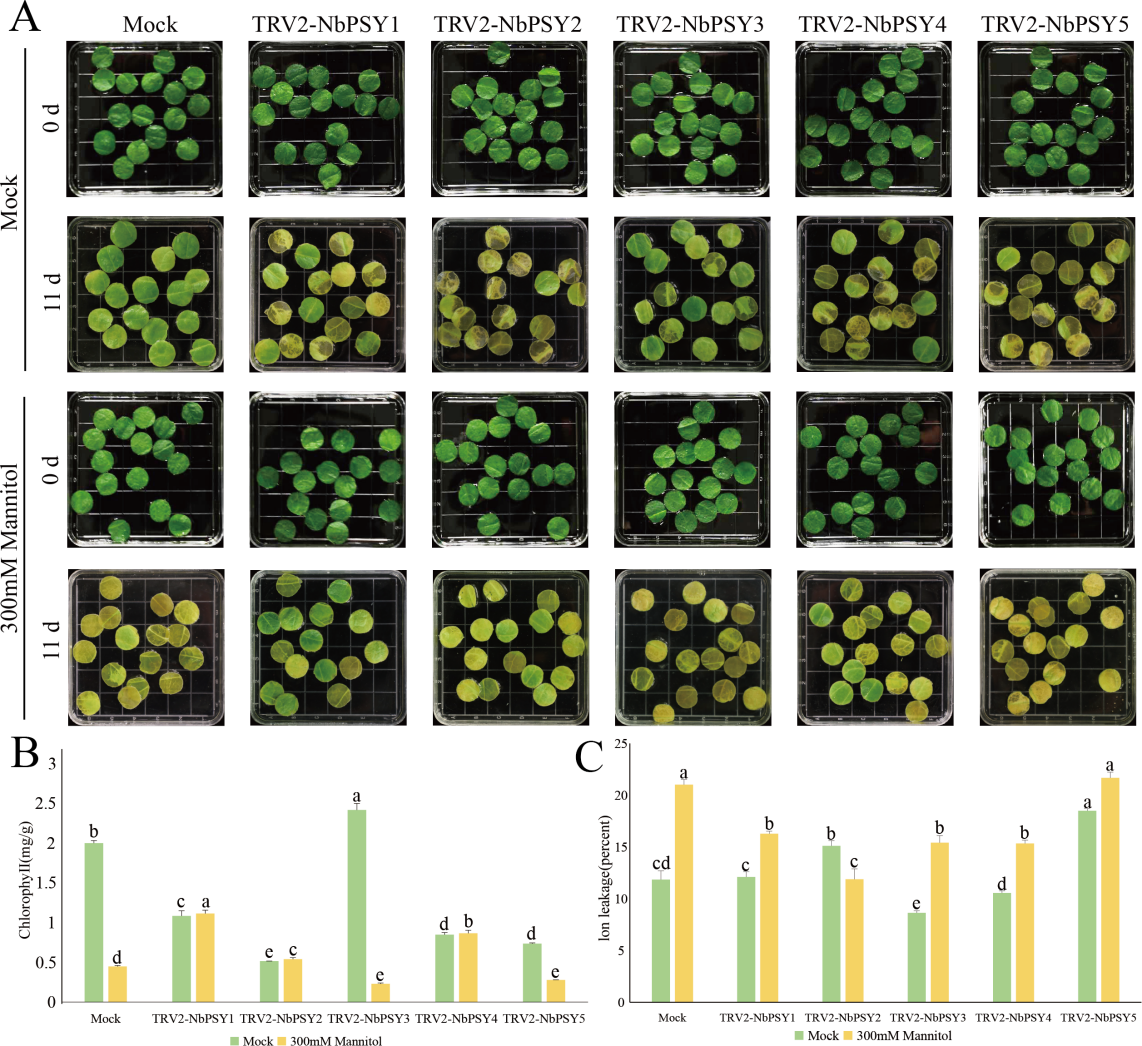
Impact of *NbPSYs* silencing on osmotic stress response. **(A)** Phenotype of *NbPSYs*-silenced detached leaves under osmotic stress. **(B)** Measurements of chlorophyll content of detached leaves. **(C)** Measurements of ion leakage of detached leaves. Data are presented as the mean + SDs of three independent experiments. Different letters above columns indicate significant differences based on Duncan’s multiple range test (P< 0.05).

Based on the expression analysis of *NtPSYs* under different stresses, we hypothesize that NtPSYs may play a crucial role in responding to osmotic stress. Therefore, we identified five *NbPSYs* in *Nicotiana benthamiana* that are similar to the conserved domains of tobacco PSYs (**Supplementary Figure S1A**). We constructed *TRV2* transient silencing vectors, and the primers and expression level after silencing are detailed in the appendix (**Supplementary Table S2**; **Supplementary Figure S1B**). Since there were no significant differences in the plants after silencing (**Supplementary Figure S1C**), we assessed the stress response of the transgenic plants by treating excised leaves and evaluating stress-induced chlorosis. After silencing *NbPSYs* the state of the detached leaves treated with water and 300 mM mannitol (**Figure 4A**) was observed. After 10 days of water treatment, the silenced leaves exhibited poorer conditions compared to the control, with lower chlorophyll content and higher ion leakage (**Figures 4B, C**). Among them, the leaves with silenced *NbPSY2* showed the lowest chlorophyll content. The increased ion leakage in *NbPSY1*, *NbPSY2*, and *NbPSY5*-silenced leaves suggests that these PSYs might play a significant role in the growth and development of *N. benthamiana*. After 11 days of mannitol treatment, leaves silenced for *NbPSY1*, *NbPSY2*, and *NbPSY4* showed less yellowing compared to the control. This was also reflected in their chlorophyll content and ion leakage rates, suggesting that NbPSYs might act as negative regulators in stress response.

### 3.7 Treatment of tobacco detached leaves with NtPSY peptides under osmotic stress

**Figure 5:**
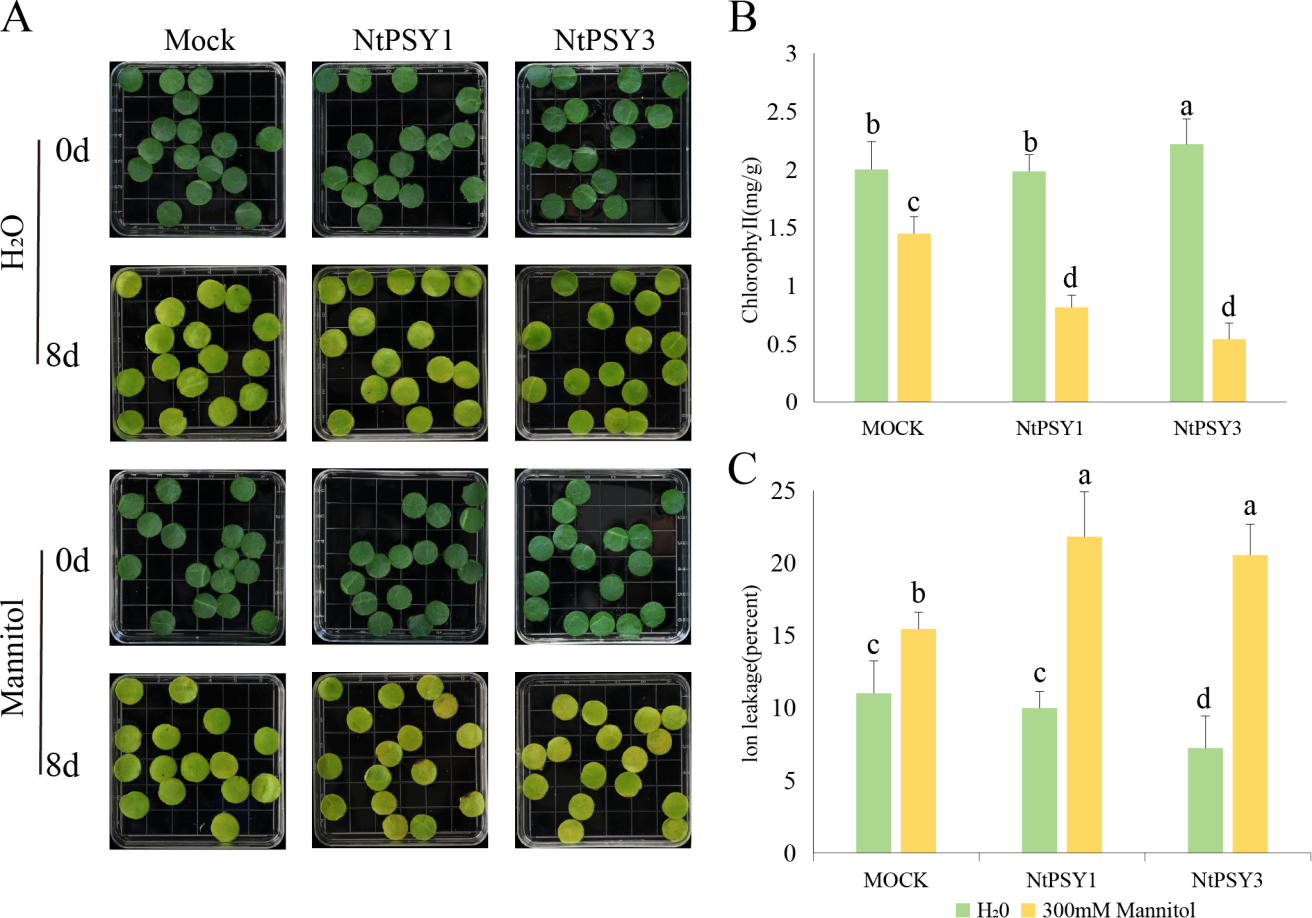
The impact of exogenous application of NtPSY peptides on leaf’s response to osmotic stress. **(A)** Tobacco detached leaves were used to test whether the synthesized PSY peptides have an impact on tobacco’s tolerance to osmotic stress. **(B)** Measurements of chlorophyll content of detached leaves. **(C)** Measurements of ion leakage of detached leaves. Data are presented as the mean + SDs of three independent experiments. Different letters above columns indicate significant differences based on Duncan’s multiple range test (P< 0.05).

Based on the phenotype exhibited by detached leaves of silenced *NbPSYs* in *N. benthamiana* under osmotic stress, we synthesized the mature peptide sequences of NtPSY1 (corresponding to NbPSY1) and NtPSY3 (corresponding to NbPSY2) *in vitro*. These peptides were then applied to detached leaves of 6-week-old K326 tobacco under osmotic stress (300 mM mannitol), at a concentration of 1µM. The results (**Figure 5A**) showed that after eight days of osmotic stress, the detached leaves treated with exogenous NtPSY1 and NtPSY3 exhibited significant yellowing compared to the control, indicating that the exogenous addition of NtPSY peptides mimicking overexpression decreased the tobacco leaves’ tolerance to stresses. This process, involving NtPSY1 and NtPSY3, can significantly accelerate senescence of leaves induced by osmotic stress, which is related to the degradation of chlorophyll and the chlorophyll-protein complex. Therefore, we measured the chlorophyll content to reflect the yellowing phenotype of the leaves. The results indicated that the chlorophyll content in leaves treated with exogenous PSY peptides was significantly higher than that in the control group (**Figure 5B**). We used ion leakage as an indicator of stress tolerance of the leaves, finding that ion leakage in the treated group was significantly higher than that in the control (**Figure 5C**).

### 3.8 Treatments of tobacco plants with NtPSY peptides under osmotic stress

**Figure 6:**
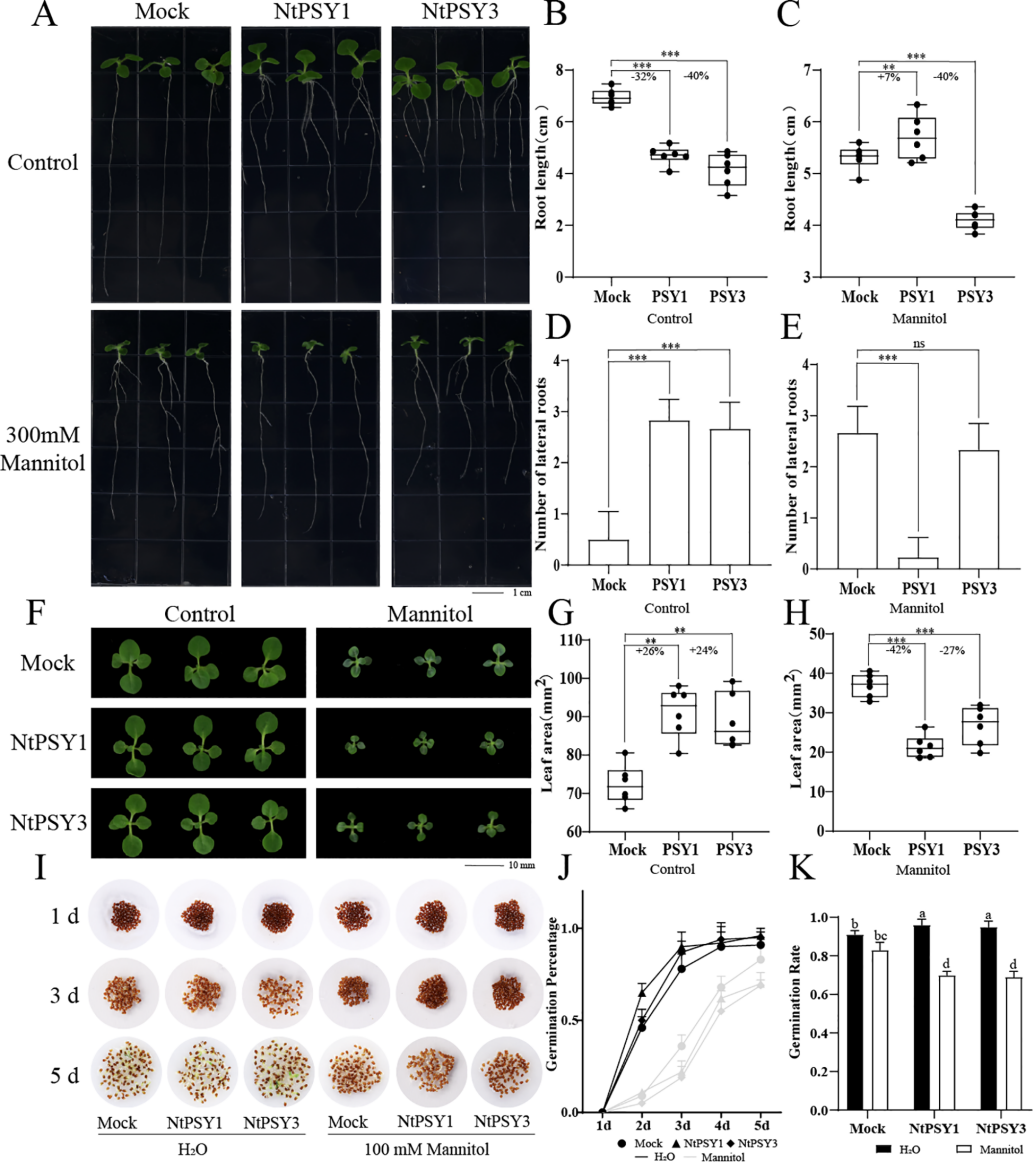
Effects of NtPSY peptides on the growth of tobacco plants and seed germination under osmotic stress conditions. **(A)** Influence of NtPSY peptides on the root length and lateral root number of tobacco seedlings. After germination, seedlings with similar root lengths were treated with 1 μM NtPSY peptide and 300 mM for 7 days. Representative images display the length of the primary root and the number of lateral roots in tobacco treated with NtCEP peptide and 300 mM. **(B)** Identification of the primary root length of tobacco under control conditions. **(C)** Identification of the primary root length of tobacco under mannitol-induced osmotic stress. **(D)** Identification of the number of lateral roots of tobacco under control conditions. **(E)** Identification of the number of lateral roots of tobacco under mannitol-induced osmotic stress. **(F)** Effects of NtPSYs on the aerial parts of tobacco seedlings. **(G)** Identification of the leaf area of tobacco seedlings under control conditions. **(H)** Identification of the leaf area of tobacco seedlings under osmotic stress conditions. **(I)** NtPSYs promote seed germination under control conditions but inhibit it under osmotic stress conditions. **(J)** Germination percentage of tobacco seeds subjected to osmotic stress. **(K)** Germination rate of tobacco seeds subjected to osmotic stress (Duncan’s multiple range test). Error bars represent the standard error of the mean (SEM) with n = 3. P< 0.01 (**), and P< 0.001 (***), according to Student’s t-test. “ns” indicates “no significance” (p > 0.05 by Student’s t-test) for the corresponding comparisons.

Finally, we treated tobacco K326 seedlings with NtPSY peptides under osmotic stress conditions, assessing the effects of the peptides on root length, lateral root number, and leaf area of the aerial parts under both control and stress conditions. Uniformly growing tobacco seedlings were transferred to MS solid media with different treatments, and their phenotypes were observed after 7 days. As shown in **Figure 6A**, under normal conditions, the addition of exogenous PSY peptides reduced the main root length of the tobacco seedlings and increased the number of lateral roots. Following the addition of NtPSY1 peptide, root length decreased by 32% compared to the control, and with the NtPSY3 peptide, root length decreased by 40% relative to the control (**Figures 6A, B**). The number of lateral roots of peptide-treated plants was higher than that of the control, with tobacco seedlings treated with PSY peptides generally having 3-4 lateral roots, whereas the control group had none (**Figure 6D**). Under osmotic stress, the root length of seedlings treated with PSY1 peptides showed an opposite phenotype, with root length 7% greater than the control, and a reduction in the number of lateral roots. The root length of the group treated with NtPSY3 peptides appeared to be unaffected by stress, consistent with treatments without stress in terms of root length and lateral root number. Compared with the control group, the root length was reduced by 40%; however, no significant difference was observed in the number of lateral roots (**Figures 6C, E**).

Regarding the phenotype of the aerial parts of the tobacco seedlings (**Figure 6F**), under control conditions, the exogenous addition of PSY peptides promoted the growth of the aerial parts, increasing leaf area. Compared to the control, the leaf area of tobacco seedlings was increased by 26% after the addition of NtPSY1 peptides, and increased by 24% with NtPSY3 peptides (**Figure 6G**). Under osmotic stress, the exogenous addition of PSY peptides inhibited the growth of tobacco seedlings; leaf area was decreased by 42% with NtPSY1 and by 27% with NtPSY3 peptides (**Figure 6H**).

Considering both above- and below-ground phenotypes, we speculate that under normal conditions, the exogenous addition of PSY peptides may cause an increase in the number of lateral roots, which facilitates the absorption of more nutrients and water, thus promoting the growth of the aerial parts and increasing leaf area. However, under osmotic stress, the exogenous addition of PSY peptides showed insensitivity in the roots, not responding to osmotic stress. After the addition of NtPSY1 peptides, there was an increase in main root length and a reduction in lateral roots. The performance with NtPSY3 peptide treatment is consistent with roots not treated with mannitol, ultimately leading to poor development of the aerial parts and reduced leaf area.

### 3.9 Treatments of tobacco seeds with NtPSY peptides under osmotic stress

In order to further investigate the role of NtPSYs under osmotic stress, we tested their effects on the germination rate of tobacco K326 seeds under osmotic stress conditions. The results showed that under osmotic stress, both the speed of germination and the germination rate of tobacco seeds were lower than those of the control group. However, tobacco seeds treated with exogenous NtPSY1 or NtPSY3 peptides displayed different phenotypes compared to the control group (**Figure 6I**). Under control conditions, the addition of either NtPSY1 or NtPSY3 accelerated seed germination, with the germination time of the control group significantly reduced in the early stages of germination. Conversely, under mannitol stress conditions, the addition of peptides resulted in a slower germination speed and ultimately a reduced germination rate (**Figures 6J, K**).We hypothesize that NtPSYs can promote seed germination under normal conditions, but under osmotic stress, NtPSYs delay seed germination to mitigate the impacts induced by stress.

## 4 Discussion

Earlier studies have shown that the PSY gene family plays a crucial role in promoting root growth and regulating the balance between plant growth and stress responses (Ogawa-Ohnishi et al., 2022). Although the PSY genes have been documented in various species, their identification and functional characterization in tobacco have not been fully explored. In this study, we used bioinformatics methods to identify 9 *NtPSY* genes in the tobacco genome and investigated their potential roles in tobacco development and stress responses. Through transcriptome data analysis, qRT-PCR experiments, and the exogenous application of synthetic NtPSY peptides, we provide the first evidence in tobacco that these peptides may serve as regulatory signals in growth and stress responses. These findings reveal the potential application value of PSY peptides in plant physiological processes and offer new insights into the molecular mechanisms of stress resistance in tobacco.

Based on phylogenetic analysis the tobacco PSY were divided into two subfamilies (**Figure 1A**), primarily distinguished by their signal peptide sequences (**Figure 2C**), reflecting the diversity of signal peptides among tobacco PSY peptides. Signal peptides are essential for protein localization and secretion, indicating that tobacco PSY family members may have distinct mechanisms of action both within and outside the cell. Protein sequence analysis revealed that, except for NtPSY6, all other tobacco PSY genes possess typical signal peptides, consistent with the sequences of PSY peptides in other plant species. This suggests that despite some sequence variation, tobacco PSY peptides may function similarly to PSY family members in *Arabidopsis* or other plants. Further analysis showed that NtPSYs possess a variable central region and a conserved C-terminal PSY domain, consistent with the characteristic patterns of known PSY family proteins. This feature supports its potential role in signaling processes. Moreover, the functions of peptides such as PSY, PSK, RGF, and CIF are closely associated with tyrosine sulfation modifications. These peptides contain a conserved (DY-) sequence at the N-terminus of their domain, which is critical for processing precursor peptides into mature peptides (Stintzi and Schaller, 2022). Tyrosine sulfation is therefore considered a key step in regulating the bioactivity of these peptides and may influence their roles in plant growth and stress responses. Previous studies have shown that the SBT protease family plays a crucial role in precursor peptide processing. For instance, SBT1.1 and SBT3.8 can process PSK precursor peptides (Srivastava et al., 2008; Stuhrwohldt et al., 2021), while SBT6.1, SBT6.2, and SBT3.8 can process RGF (Ghorbani et al., 2016; Stuhrwohldt et al., 2020). Based on these findings, it is hypothesized that NtPSY precursor proteins may also be processed into mature peptides through a similar mechanism by SBT proteases. Although six AtPSY mature polypeptides have been characterized in *Arabidopsis*, all starting with DY and encompassing the entire PSY domain (Ogawa-Ohnishi et al., 2022).

Cis-acting elements play a central role in gene regulatory networks, as they can sense environmental signals (such as drought, salinity, and biotic stress) and regulate the dynamic expression of genes, thereby helping plants to adapt to external stresses. To explore the potential role of NtPSYs in response to abiotic stress, we analyzed the distribution of cis-acting elements in its promoter region. The results revealed that the promoters of *NtPSYs* are enriched with stress-responsive elements, suggesting that *NtPSYs* could be important genes involved in plant responses to stress signals. RNA-Seq data from public expression databases show that *NtPSYs* are expressed in multiple tissues of tobacco, highlighting their potential significant role in tobacco growth and development. This also indirectly suggests that the function of the PSY genes may be conserved across different plant species. Notably, this widespread tissue expression pattern may reflect the multifunctionality of NtPSYs, as they likely participate in basic physiological processes and may also play a regulatory role in stress responses.

To further elucidate the function of NtPSYs, we performed qRT-PCR analysis to investigate its expression patterns under four different abiotic stresses (osmotic stress, salt stress, low-temperature stress, and high-temperature stress). The results showed that all *NtPSY* genes respond to these stresses, but exhibit differential expression patterns depending on the type of stress and time point. *NtPSY5* expression was significantly upregulated by 280-fold after 3 hours of osmotic stress treatment, suggesting that it may play a key role in the signaling pathways involved in dehydration or water deficit responses. This significant induction effect implies that NtPSY5 might enhance tobacco’s adaptation to osmotic stress by activating downstream stress-responsive genes or regulating signaling pathways. In contrast, *NtPSY4* showed a downregulation trend under osmotic stress, which may be associated with maintaining physiological balance under stress conditions, suggesting a potential antagonistic or compensatory role. Furthermore, most *NtPSY* genes exhibited insensitivity or downregulated expression under low-temperature stress, which might reflect their limited role in cold stress responses, or it may indicate that plants prioritize other pathways to cope with low-temperature stress. Under salt stress, except for *NtPSY3* and *NtPSY6*, all other *NtPSY* genes were significantly upregulated, indicating that they may be involved in maintaining ion balance or enhancing osmotic protection. Overall, the *NtPSY* genes show specific and diverse regulatory patterns under different stress conditions, suggesting that they play an important role in tobacco’s environmental adaptability. In *Arabidopsis*, *AtPSY1* and *AtPSY4* exhibit significant responses to biotic stress, especially *AtPSY1*, which shows significant upregulation under various stress conditions (Amano et al., 2007; Matsuzaki et al., 2010). This observation aligns with our findings in tobacco, suggesting a conserved function in stress signaling between the two species. Similarly, *OsPSY* genes in wheat also show upregulation under abiotic stress (Kesawat et al., 2024), further supporting the central role of the PSY gene family in regulating stress adaptation. However, differences in the regulatory patterns between species and gene members reflect their specialized evolution in stress responses. Further investigation into the downstream regulatory networks of NtPSYs and its functional differentiation mechanisms will enhance our understanding of the molecular basis of plant adaptation to stressful environments and provide theoretical support for breeding crops with improved stress tolerance.

The adaptation of plants to abiotic stresses such as drought is a key process for ensuring their survival and maintaining productivity. The above study indicates that the NtPSY genes plays an important role in plant responses to abiotic stress. To further explore their function, we applied the VIGS method to transiently silence the *NbPSY* genes in *Nicotiana benthamiana*. We found that silencing *NbPSY1*, *NbPSY2*, and *NbPSY5* resulted in a reduction in chlorophyll content and an increase in ion leakage in plant leaves under normal growth conditions, indicating that these genes play a positive regulatory role in maintaining cellular homeostasis, photosynthesis, and plant growth. However, under mannitol-induced stress conditions, leaves with silenced *NbPSY1*, *NbPSY2*, and *NbPSY4* exhibited lower degrees of chlorosis and higher chlorophyll content, suggesting that the NbPSY genes may act as negative regulators in stress response (**Figure 4**). They likely influence plant stress tolerance by inhibiting the activation of certain stress resistance pathways.

Subsequently, we synthesized NtPSY1 and NtPSY3 polypeptides artificially and treated the detached leaves similarly. These leaves developed a chlorotic phenotype and were sensitive to osmotic stress. This observation suggests that NtPSYs might negatively influence stress tolerance in tobacco leaves, potentially by interfering with the normal stress response mechanisms or by altering cellular homeostasis. The chlorotic phenotype could indicate a disruption in chlorophyll biosynthesis or degradation pathways, which are often linked to stress responses and overall plant health. We found that the exogenous application of NtPSY peptides promoted tobacco seed germination, but it reduced the germination rate under osmotic stress (**Figure 6**). Previous studies have shown that GAD1 and OsEPFL2 in rice can promote seed germination by reducing abscisic acid (ABA) levels (Jin et al., 2023). ABA can inhibit seed germination to enhance seedling survival under abiotic stress conditions (Finch-Savage and Leubner-Metzger, 2006). We hypothesize that NtPSYs, being peptide signals, may also promote tobacco seed germination in a similar manner, which may partially explains why exogenous NtPSY peptides reduce the germination rate under osmotic stress. This suggests that PSY may regulate seed adaptation to environmental stresses by downregulating related genes under such conditions. Developing optimal root morphology, including lateral root formation, is crucial for transplant growth. Lateral roots not only provide anchorage but also enhance water-use efficiency and nutrient extraction from the soil (Casimiro et al., 2001; Peret et al., 2009). Our experiments demonstrated that NtPSYs increase lateral root number and promote aerial leaf growth in tobacco seedlings. Yet, these seedlings show higher intolerance under osmotic stress. This phenomenon might be due to that NtPSYs promoting growth at the expense of stress tolerance, highlighting the complex balance between growth and stress response. Similarly, overexpression of *AtPSY6* in *Arabidopsis* promotes growth but weakens salt tolerance. This effect is mediated through the AtPSY receptor, where the binding of AtPSY reduces receptor function, thereby weakening stress resistance, primarily manifested in metabolic damage areas. Additionally, the exogenous addition of AtPSY5 can rapidly reduce the expression of stress-related genes. These stress signaling transduction networks actively suppress cellular metabolic activities, leading to severe limitations in plant growth (Ogawa-Ohnishi et al., 2022). ABA accumulation is a critical response in plants to osmotic stress, triggering diverse adaptive mechanisms (Gill and Tuteja, 2010). ABA strongly activates SNF1-related protein kinase 2.2 (SnRK2.2), SnRK2.3, and SnRK2.6, which are essential kinases for osmotic stress signaling (Fujii et al., 2009). Consequently, NtPSY may promote tobacco growth by reducing ABA levels. However, this reduction in ABA levels could ultimately render seedlings more sensitive to osmotic stress due to compromised activation of stress-responsive pathways. These results indicate that the PSY genes are highly conserved in plants’ responses to various stress conditions, while highlighting the important role of PSY peptides in regulating growth and stress responses. In summary, PSY genes and their peptides exhibit versatility in plant growth and stress adaptation: they not only promote growth and development but also potentially regulate stress responses through complex signaling networks. The trade-off between growth and stress tolerance provides a potential strategy for improving crop performance in the future through the regulation of the PSY pathway.

Additionally, there are certain limitations to the bioinformatics approaches used in this study. For instance, the incomplete genomic data of tobacco may lead to the loss of critical information regarding the chromosomal location of PSY, and the predictive nature of bioinformatics tools may result in inaccuracies between predicted protein functions and their biological realities. While we have gained some insights into the role of NtPSYs in tobacco, further molecular validation is needed. This includes measuring malondialdehyde (MDA) content and superoxide dismutase (SOD) activity in detached leaves after stress treatments, detecting stress-related marker genes, and assessing hormone levels during seed germination.

## 5 Conclusions

In this study, we identified nine members of the tobacco PSY gene family and conducted bioinformatics analysis of these genes, including their gene structure, conserved domains, and expression patterns under different stresses. Using VIGS, we preliminarily determined the function of PSYs under osmotic stress. Further studies suggest that the synthesized tobacco PSY peptides play a crucial role in mediating the trade-off between tobacco growth and stress responses. Specifically, under optimal conditions, they promote seed germination, lateral root formation, and overall tobacco growth, while under stress treatments, they reduce plant resistance to stresses. This mechanism balances stress tolerance with the associated energy costs, allowing the plant to achieve optimal growth under stressful environmental conditions. Furthermore, the PSY receptor in tobacco has yet to be identified, indicating a gap that requires further research to elucidate the antagonistic relationship between PSY-mediated stress adaptation and growth. By studying NtPSY mutants and overexpression transgenic plants, we can better understand its specific mechanisms. Thus, future research will focus on the role of NtPSY mutants. In summary, our work not only provides valuable insights for future functional analyses of PSY in tobacco but also offers practical significance for agricultural applications, such as developing stress-tolerant tobacco varieties, which will contribute to the broader field of plant stress physiology.

## Data Availability

The original contributions presented in the study are included in the article/**Supplementary Material**. Further inquiries can be directed to the corresponding author.

## Supplementary material

Supplementary Figure 1 Conservation domain sequences of NtPSYs and NbPSYs and the phenotype of Nicotiana benthamiana silencing.
