# Supplementary material for: Tobacco (Nicotiana tabacum) PSY peptides and their potential roles in seed germination, vegetative growth, and leaf senescence under osmotic stress
Source: Front Plant Sci. 2025 May 28;16:1575308. doi: 10.3389/fpls.2025.1575308 (PMC12153210; doi:10.3389/fpls.2025.1575308)
Supplement: Supplementary Figure 1 — Conservation domain sequences of NtPSYs and NbPSYs and the phenotype of Nicotiana benthamiana silencing. [file DataSheet1.docx]

Supplementary Material

**Table S1:** Primers used for real-time PCR analysis

| Gene name | Forward primer sequences | Reverse primer sequences |
| --- | --- | --- |
| *NtActin* | GAAGAAGGTCCCAAGGGTTC | TCTCCCTTTAACACCAACGG |
| *NtPSY1* | GGTCAATGACTATCCAGGTTCT | CCATAACTCAGCTACGGACATA |
| *NtPSY2* | AGCTGTGTCGAATGCTAAGATT | AGTTAGCTTCTCCTCTCTCAGA |
| *NtPSY3* | GACGCTGAGCTCAATGATTATC | TTCTCGATTTGTGCATGTCATC |
| *NtPSY4* | GGTAAAGTCACTAAAGTTCGCG | GCTATCCCTCTCTCCTCTTCTA |
| *NtPSY5* | AAGCCTCAAGACAATTACAGGA | TAGCACCTGTTCCATCATAGTC |
| *NtPSY6* | CCACCATTGCAGTTCATTACTC | TCCACCATAGTCATCTCTCTCA |
| *NtPSY7* | AGAGATGACTATGCAGGAACAG | TACATCTACTTCACTGCACACA |
| *NtPSY8* | CCTGCAGATGGTAAATTGCTAC | CCAAAACTCAGCAACATCTCAA |
| *NtPSY9* | GTTGATGATGCTAGATGCTGTG | AACTCATTGGAAGCTCTGCTAT |

**Table S2:** Primers used for VIGS

| Gene name | Forward primer sequences | Reverse primer sequences |
| --- | --- | --- |
| NbPSY1-OE | ATGGCTGGCCCTTTCTTTCATT | TTAGCATTCGGCACAGCCTCTC |
| NbPSY2-OE | ATGGCTGCCACTTCTCTAACTT | TAAGGGTGGCCTGGTGTGTGGT |
| NbPSY3-OE | ATGGAGCACAAGGCGTCCTTCCT | CTAGTCGCCTCCAGGAGGCGAT |
| NbPSY4-OE | ATGGAGCACAGGTCTTCTAATTTA | TCATTCAGTCCCAGGAGGTTTTG |
| NbPSY5-OE | ATGAGACCAATCAAAGCTTGTCT | TCAAGGGTGATTGCCACCTCTC |
| NbPSY1-TRV2 | AAGGTTACCGAATTCATGGCTGGCCCTTTCT | CGTGAGCTCGGTACCTTAGCATTCGGCACA |
| NbPSY2-TRV2 | AAGGTTACCGAATTCATGGCTGCCACTTCTC | CGTGAGCTCGGTACCTAAGGGTGGCCTGGT |
| NbPSY3-TRV2 | AAGGTTACCGAATTCATGGAGCACAAGGCG | CGTGAGCTCGGTACCCTAGTCGCCTCCAGG |
| NbPSY4-TRV2 | AAGGTTACCGAATTCATGGAGCACAGGTCT | CGTGAGCTCGGTACCTCATTCAGTCCCAGG |
| NbPSY5-TRV2 | AAGGTTACCGAATTCATGAGACCAATCAAA | CGTGAGCTCGGTACCTCAAGGGTGATTGCC |


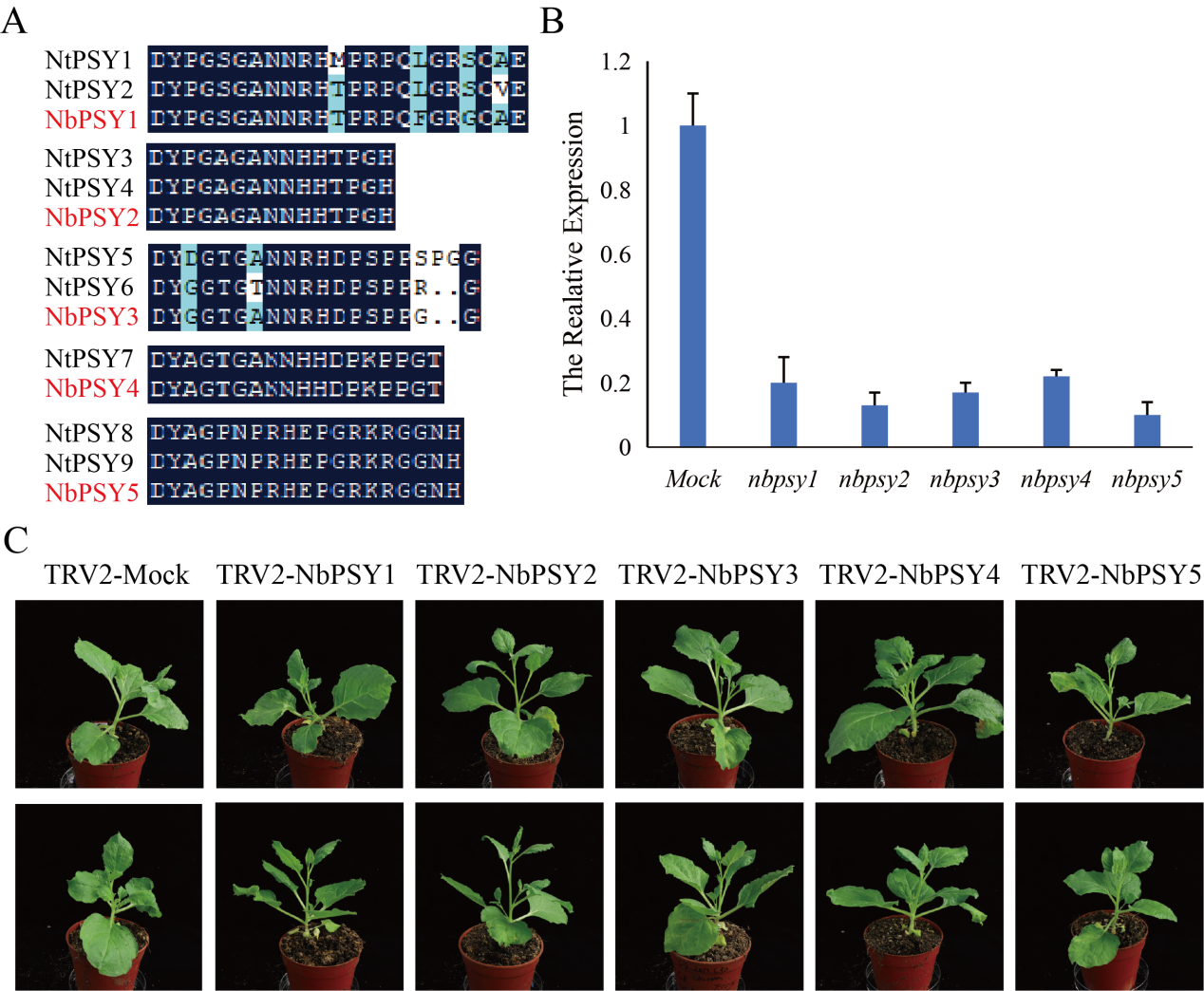


Figure S1: Conservation domain sequences of NtPSYs and NbPSYs and the phenotype of Nicotiana benthamiana silencing. A, Multiple sequence alignment of conserved structural domain sequences of NtPSY1-9 and NbPSY1-5 PSY proteins. B, VIGS gene silencing expression identification in Nicotiana benthamiana. C, Phenotypic changes of Nicotiana benthamiana plants after VIGS gene silencing.
